# Scintillation characteristics of chemically processed Ce:GAGG single crystals

**DOI:** 10.1371/journal.pone.0281262

**Published:** 2023-03-07

**Authors:** Chansun Park, Sangsu Kim, Alima Melis, Wonhi Lee, Abdallah Elmughrabi, Shinhaeng Cho, Jung-Yeol Yeom

**Affiliations:** 1 BK21 Four R&E Center for Precision Public Health, Korea University, Seoul, Republic of Korea; 2 School of Biomedical Engineering, Korea University, Seoul, Republic of Korea; 3 Interdisciplinary Program in Precision Public Health, Korea University, Seoul, Republic of Korea; 4 Global Health Technology Research Center, Korea University, Seoul, Republic of Korea; 5 Department of Bioengineering, Korea University, Seoul, Republic of Korea; 6 Department of Bio-Microsystem Technology, Korea University, Seoul, Republic of Korea; 7 Department of Radiation Oncology, Chonnam National University Medical School, Gwangju, Republic of Korea; University of Salento, ITALY

## Abstract

We investigated the correlation between the surface finish and luminescence properties of chemically polished cerium-doped single-crystal Gd_3_Al_2_Ga_3_O_12_ scintillators (Ce:GAGG), from the crystallographic perspective. The intrinsic defects in the crystals were identified via photoluminescence spectroscopy followed by scanning electron microscopy and X-ray diffraction to analyze their surface morphologies. Finally, the samples were individually wrapped with an enhanced specular reflector (ESR), coupled with a photomultiplier tube, placed inside a dark box, connected to a digitizer, and irradiated with a ^137^Cs radioactive source to evaluate the relative light (signal) output and energy resolution of each sample. The as-cut (rough) Ce:GAGG single-crystal samples, that were chemically polished with phosphoric acid at 190°C in air for 60 min, demonstrated a 33.1% increase in signal amplitude (light output to photosensor) and 2.4% (absolute value) improvement in energy resolution, which were comparable to those obtained for the mechanically polished sample. For these samples, the surface roughness was found to be ~430 nm, which was approximately half of that of the mechanically polished sample. The chemical polishing method used in this study is a cost-effective and straightforward technique to improve structural imperfections and can facilitate the treatment of inorganic scintillators with complex shapes and/or on a large scale.

## Introduction

Radiation detection and monitoring are gradually emerging as an integral part of numerous routine applications, including medicine, high-energy physics, homeland securities, and oil well logging, and inorganic scintillators that convert radiation into scintillation light are steadily becoming instrumental components of radiation detectors and monitors [1]. The conversion of radiation into scintillation light and its subsequent detection using photodetectors is strongly dependent on the intrinsic quality and fabrication of the scintillator. Thus, a significant improvement in the optical and luminescence properties of inorganic scintillators is crucial for developing highly efficient scintillators for the aforementioned applications [2]. Since 2010, several promising candidate materials, doped with rare earth elements that exhibit attractive properties, such as high density, fast timing response, and high optical transparency, have been introduced [3, 4]. Among such candidate materials, cerium-doped Gd_3_Al_2_Ga_3_O_12_ (Ce:GAGG) has gained widespread attention owing to its high light yield [5–7]. In addition, the Ce:GAGG scintillator with the garnet structure is a non-hygroscopic material with a density of 6.63 g/cm^3^ and a light yield of 56,000 photons/MeV [7].

The light yield of a scintillator is affected by its crystallographic structure; moreover, inevitable microscopic conditions, such as surface roughness and defects produced near the surface during the manufacturing (e.g., dicing), can reduce the scintillation light output. Therefore, to obtain superior optical and luminescence properties, the crystallographic structure is modified via additional post-crystal-growth treatments, such as thermal annealing and surface finishing [8]. Because the surface of the crystal contains defects and impurities generated from the crystal growth or surface finishing, they can affect scintillation properties (luminescent and optical transport) of the crystals.

In addition, scintillation light photons undergo multiple transmissions, reflections, absorptions, and scatterings during their propagation to the photodetector, leading to light loss, which affects the total light collection efficiency of the photodetector such as photomultiplier tube (PMT) and semiconductor detectors. Rough and smooth surfaces are known to exhibit vastly different reflection characteristics—the former leads to diffused reflections, whereas the latter causes specular reflections.

The above-mentioned factors imply that the surface finish of a scintillator plays a crucial role in its scintillation and light-collection efficiencies. Several authors have reported various surface finishing techniques to mitigate the heterogeneity of a rough scintillator-crystal surface [9–16]. According to these reported studies, employing improved polishing or coating techniques can aid in reducing the surface damage or surface inhomogeneity produced during the crystal growth, and improve the light transmission efficiency of a scintillator crystal. For rectangular scintillators, mechanical polishing is widely employed to flatten the surfaces, resulting in crystals with uniform surfaces. However, polishing irregularly shaped scintillators, such as cylindrical and curved scintillators, or small-sized scintillators using conventional mechanical polishing techniques can be a considerable challenge [17–19]. Therefore, alternate versatile polishing methods are required for producing homogeneous crystal surfaces. Consequently, in a previous study, we evaluated the feasibility of using chemical polishing as a potential method for creating uniform ceramic scintillator surfaces and investigated the effect of chemical polishing time on the surface microstructure of a ceramic scintillator [20]. Because ceramic scintillators are often brittle and relatively translucent compared to their single-crystal (monocrystalline) counterparts, their use in radiation detection is typically limited to relatively thin detectors.

Chemical polishing of single crystals is a cost-effective polishing method, particularly when complex-shaped scintillators are involved. Slates et al. [11] reported that chemically polishing a lutetium oxyorthosilicate (LSO) scintillator by dipping in phosphoric acid increases its light output by 250% compared to unpolished crystals. Further, in terms of the depth-of-interaction resolution, an improved light collection can be achieved by etching an LSO scintillator for 5 min, as demonstrated by Shao et al. [15]. Moreover, surface etching or polishing using chemicals has been demonstrated to be an effective method for rearranging the surface topography of scintillators to obtain smooth surfaces for radiation detection applications [21, 22].

The present study was primarily performed to investigate the effect of chemical polishing of Ce:GAGG single crystals on their light outputs for different treatment times and crystal thicknesses. Tools such as photoluminescence (PL), energy-dispersive X-ray spectrometry (EDS), X-ray diffraction (XRD) and were used to assess the impact of surface modification on the scintillation properties of the crystal from the crystallographic perspective, and surface profiler to measure morphological changes.

Although several chemical etchants, such as acetic acid, nitric acid, citric acid, hydrofluoric acid, and phosphoric acid, are available for chemical polishing, we used phosphoric acid for treating the Ce:GAGG samples, similar to our previous study, because it is less reactive, cost effective, and easily available. The investigation of the correlation between the chemical polishing duration/temperature and Ce:GAGG crystal thickness is significant for radiation detection applications, where scintillators of various thicknesses are used, such as nuclear medicine.

## Materials and methods

### Sample preparation and polishing

**Fig 1:**
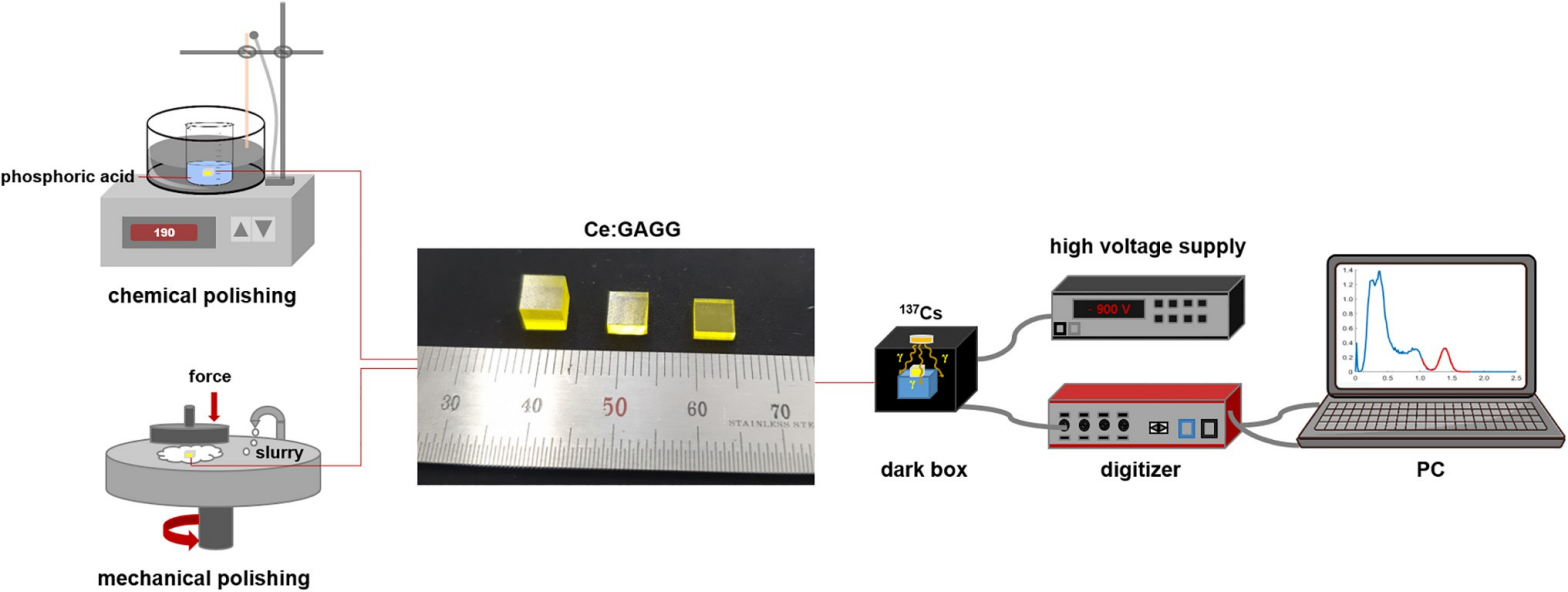
Schematic of the sample surface treatments and spectral data acquisition.

To evaluate the spectral performance, we prepared 5 × 5 mm^2^ unpolished as-cut samples of varying thicknesses (2, 5, 10, and 20 mm) sawed from a larger (5 × 5 × 50 mm^3^) unpolished Ce:GAGG single-crystal block (TPS, Republic of Korea) grown by the Czochralski technique. The mechanically polished reference samples were prepared using a polisher (XP 8, Ted Pella) with alumina suspension (particle size: 1 μm). The chemically polished samples were fabricated by dipping the as-cut samples into a beaker containing phosphoric acid (85% in volume), placed in a silicone oil bath, to evaluate the effect of chemical polishing on the light output of the crystal samples [11, 12]. First, to investigate the correlation between the chemical polishing time and the etching rate, groups of 5 × 5 × 2 mm^3^ Ce:GAGG single-crystal samples were etched separately for 5, 10, 20, 30, 60, 90, and 120 min. As described before, the transmittance of the scintillation light to the photosensor is also dependent on the crystal thickness; thus, the Ce:GAGG single-crystal samples of 2, 5, 10, and 20 mm thicknesses were chemically polished for 30, 60, 90, and 120 min to compare their light outputs. The two polishing methods and spectral data acquisition process are illustrated in Fig 1. After the chemical polishing, each sample was cleaned with deionized water and dried in air. Next, the change in the weight of each sample was recorded to assess the weight loss due to chemical polishing.

### Evaluation of spectral performances

Each sample was coupled to a PMT (Hamamatsu, H11934-100) using optical grease (BC-630, Saint-Gobain) to evaluate its spectral performance. The detector system was kept inside a dark box to prevent unwanted stray light and was irradiated by a ^137^Cs radioactive source. A reflector cap was formed by folding an enhanced specular reflector (3M) sheet to collect the light photons generated in the scintillator, and all the samples were covered with the same reflector to eliminate the dependence of the energy spectrum measurement on the type and thickness of the reflectors. A high voltage supply (Ortec 556, Ametek) was used to provide a fixed voltage of -900 V to the PMT, and the PMT output signal was sent to a digitizer (CAEN, DT5730). The acquired γ-ray waveforms were recorded in a computer for computation as follows: the signal peaks were initially identified by setting an arbitrary threshold. Then, from the located peak position, two points (limits of integration), each 1% from the baseline at the trail and leading edges, were located for integration. The integral of the waveforms was used to plot the energy spectrum, from which the energy resolution and relative light output of each sample were calculated at the position of the 662 keV photopeak of ^137^Cs via Gaussian fitting of the energy histogram.

### Inspection of structural and optical properties

The surface changes were evaluated via roughness measurements using a surface profilometer (Bruker, DektakXT Stylus Profiler). For each sample, five different areas were selected on the 5 × 5 mm^2^ face of the sample, with a verified range of 2 μm, and the average values of the roughness and waviness were used for representing the final surface roughness and waviness. The microstructures of the Ce:GAGG sample surfaces were investigated using scanning electron microscopy (SEM; SEC’s SNE-4500M Tabletop SEM with 5-Axis Stage Control). For the light transmission measurements, a UV–Vis–NIR spectrometer (Cary 5000, Agilent Technologies) was used with the following parameters: wavelength range of 200–800 nm, scan rate of 600 nm/min, data resolution of 1 nm, and averaging time of 0.1 s. The photoluminescence (PL) spectra were obtained using a confocal Raman spectrometer (LabRam Aramis, Horriba Jovin Yvon) with 325 nm He–Cd laser excitation at room temperature. The generated PL is from the band-to-band excitonic recombination stimulated by the light, and it can be one of the evaluations of the crystallinity. The crystallinity and composition of the crystals were also assessed via X-ray diffraction (XRD; Smartlab, Rigaku) analysis in the range of 10°–90° using Cu-Kα radiation (λ = 1.54056 Å). The changes in the chemical composition after different surface treatments were measured by energy-dispersive X-ray spectrometry (EDS; JEOL JSM-7610FPlus).

## Results and discussion

### Optical and luminescence measurements

PL measurement is a nondestructive method for investigating the electronic band excitation and relaxation processes in a medium [23]. Compared with a smooth polished surface, a rough scintillator surface contains several discontinuities in the crystallographic structure and optical barriers, such as grains, grain boundaries, and dangling bonds. These defects become the sources of non-radiative emissions, which degrade the scintillation performance of a scintillator [24]. Overall, the competition between these non-radiative and radiative processes determines the luminescence characteristics of a scintillator crystal. In other words, the PL intensity measurements can be an initial yet helpful method for revealing the post-treatment surface conditions.

**Fig 2:**
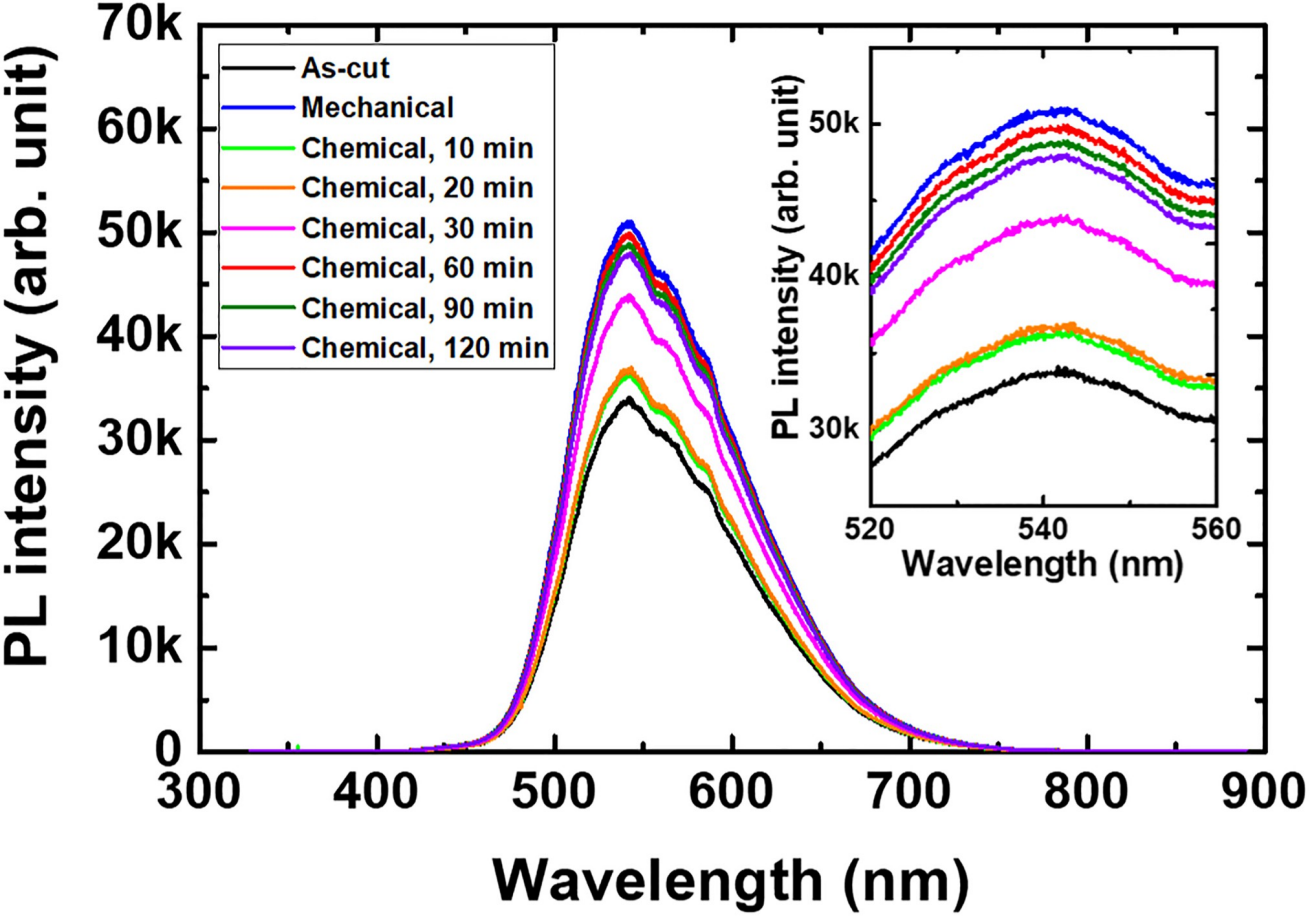
PL emission spectra emitted by the 5 × 5 × 2 mm^3^ Ce:GAGG single-crystal samples under the 325 nm He–Cd laser excitation: (a, black) as-cut, (b, blue) mechanically polished, (c)–(h) chemically polished for ((c, neon green), (d, orange), (e, magenta), (f, red), (g, green), (h, violet)), for 10, 20, 30, 60, 90, and 120 min.

The PL spectra shown in Fig 2 are indicative of the relationship between the surface treatment and the relative emission intensity [23, 25]. Evidently, the mechanically polished sample exhibited the highest PL intensity. In contrast, increasing the chemical polishing time resulted in a PL intensity enhancement up to 60 min; further treatment beyond this time did not contribute to the luminescence enhancement. Instead, a longer chemical polishing time, exceeding 60 min, led to an excessive scintillator weight (material) loss.

To elucidate the effect of the chemical processing on the crystal samples, changes on the crystal surfaces after each surface treatment are summarized in Table 1. Chemical polishing produces morphological changes and alters the elemental composition of the crystals at the surface. In this study, when the samples were immersed in phosphoric acid, the atoms on the surface were selectively removed during the reaction with H_3_PO_4_, as evident from the results presented in Table 1. This reaction reduced the number of dangling bonds, thereby improving the luminescence properties of the scintillator. This is also visible in the SEM image where the surface acquires regular facets with chemical polishing (rather than random roughness) likely indicating selective removal of atoms through anisotropic orientation-dependent acid etching at the surface.

**Table 1:** EDS analysis results of the Ce:GAGG crystal samples after different surface treatments. Each value denotes an average value obtained from measurements over five different spots on the samples.

Treatment	Elemental composition (wt%)					
	O	Al	P	Ga	Ce	Gd
As-cut	28.6	7.5	0.0	22.5	0.0	41.4
Mechanical	24.8	7.8	0.0	21.4	0.0	46.0
Chemical, 10 min	22.9	7.5	0.1	21.0	0.0	48.5
Chemical, 20 min	19.3	7.9	0.2	20.4	0.0	52.2
Chemical, 30 min	16.6	8.0	0.2	18.9	0.0	56.3
Chemical, 60 min	15.1	6.8	0.3	15.7	0.0	62.1
Chemical, 90 min	15.4	7.2	0.2	16.0	0	61.2
Chemical, 120 min	15.2	6.9	0.3	16.8	0	60.8

**Fig 3:**
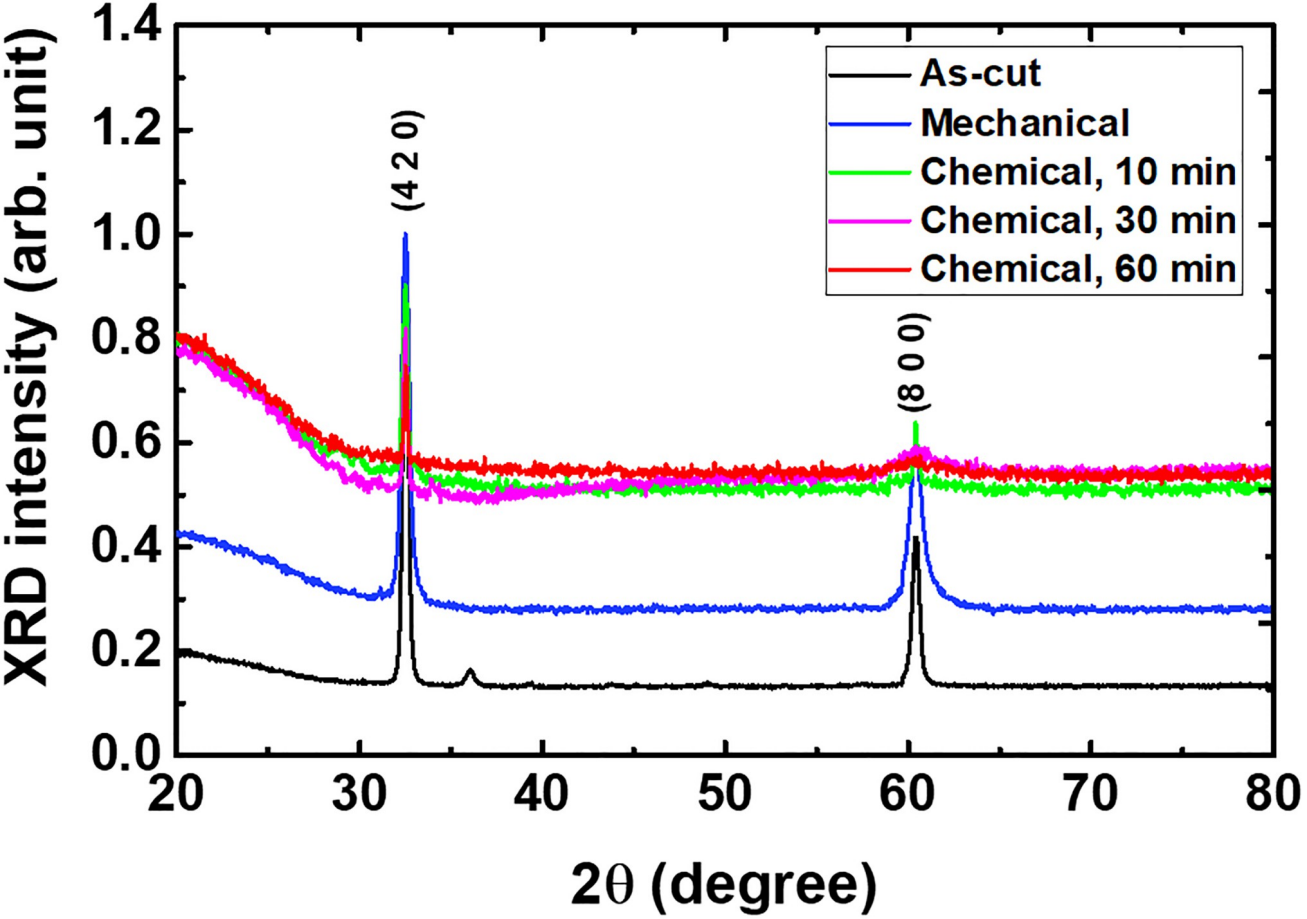
XRD spectra of the Ce:GAGG samples after different surface treatments, showing peaks at: (a) ~ 32.5° and (b) ~ 60.4°.

Phase identification near the scintillator surface was performed using XRD to reveal the crystallinity (atomic structure) after the different surface treatments, as shown in Fig 3. The different polishing methods (both mechanical and chemical polishing) resulted in changes in the XRD peak intensities of Ce:GAGG single crystals. Specifically, the chemically and mechanically polished Ce:GAGG samples exhibited changes in XRD peaks compared to the unpolished sample. Considering PL and EDS data together, the crystallinity at the surface likely improved with chemical treatment.

**Fig 4:**
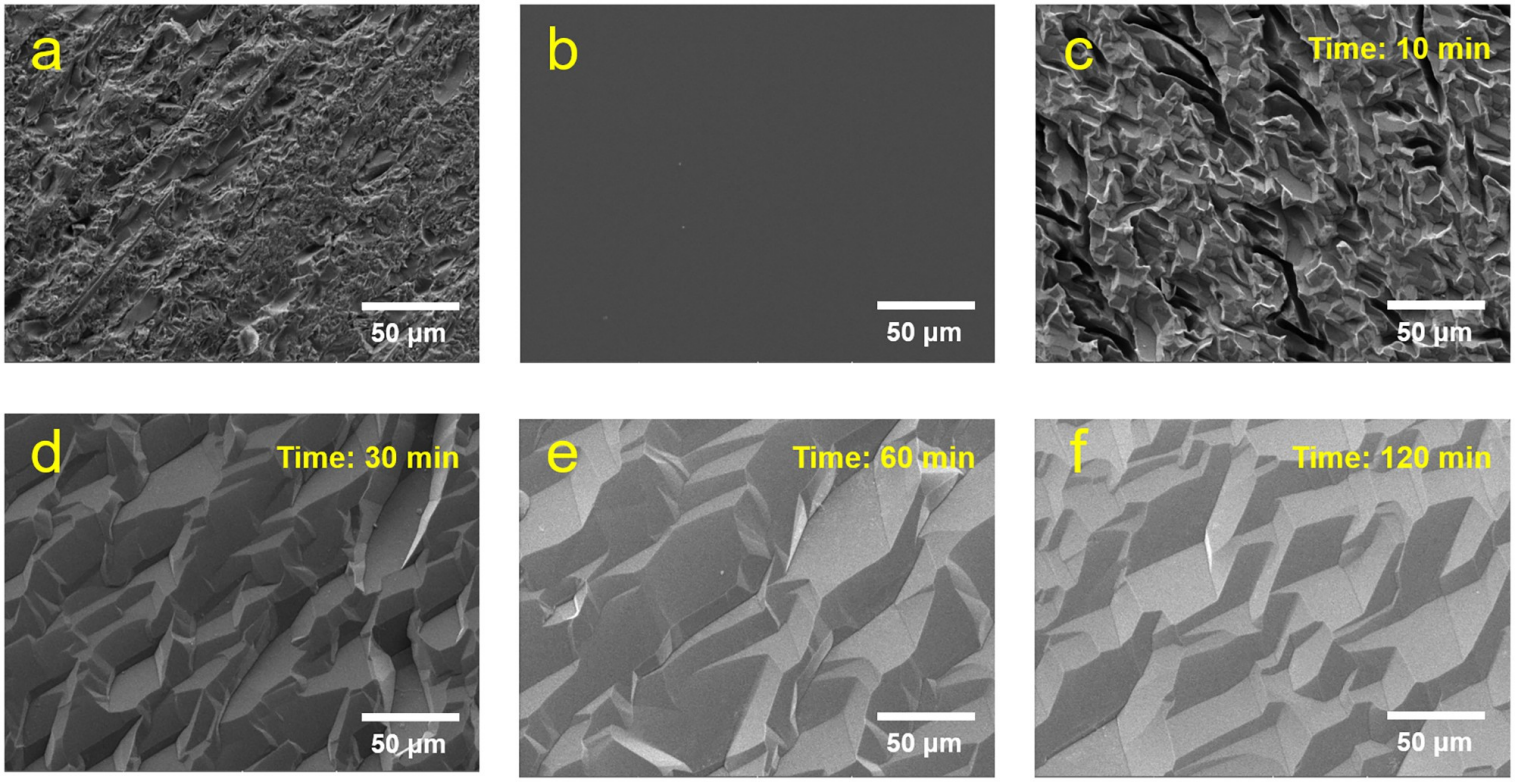
SEM images of the 5 × 5 × 2 mm^3^ Ce:GAGG single crystals: (a) as-cut, (b) mechanically polished, and (c)–(f) chemically polished for 10, 30, 60, and 120 min.

The sample surfaces are visible in the SEM images shown in Fig 4. These images were acquired at a scale of 50 μm (horizontal field-of-view) to observe the changes after different surface treatments.

As shown in Fig 4(a), the surface inhomogeneities are evident before the mechanical and chemical treatments; however, they were smoothed out by the surface deformations that occurred after the surface treatments. When the immersion time was increased to 60 min, the Ce:GAGG crystal surface topography was rearranged, and the surface delineation was blurred, along with a decrease in the number of surface defects, as shown in Fig 4(c)–(4f).

**Fig 5:**
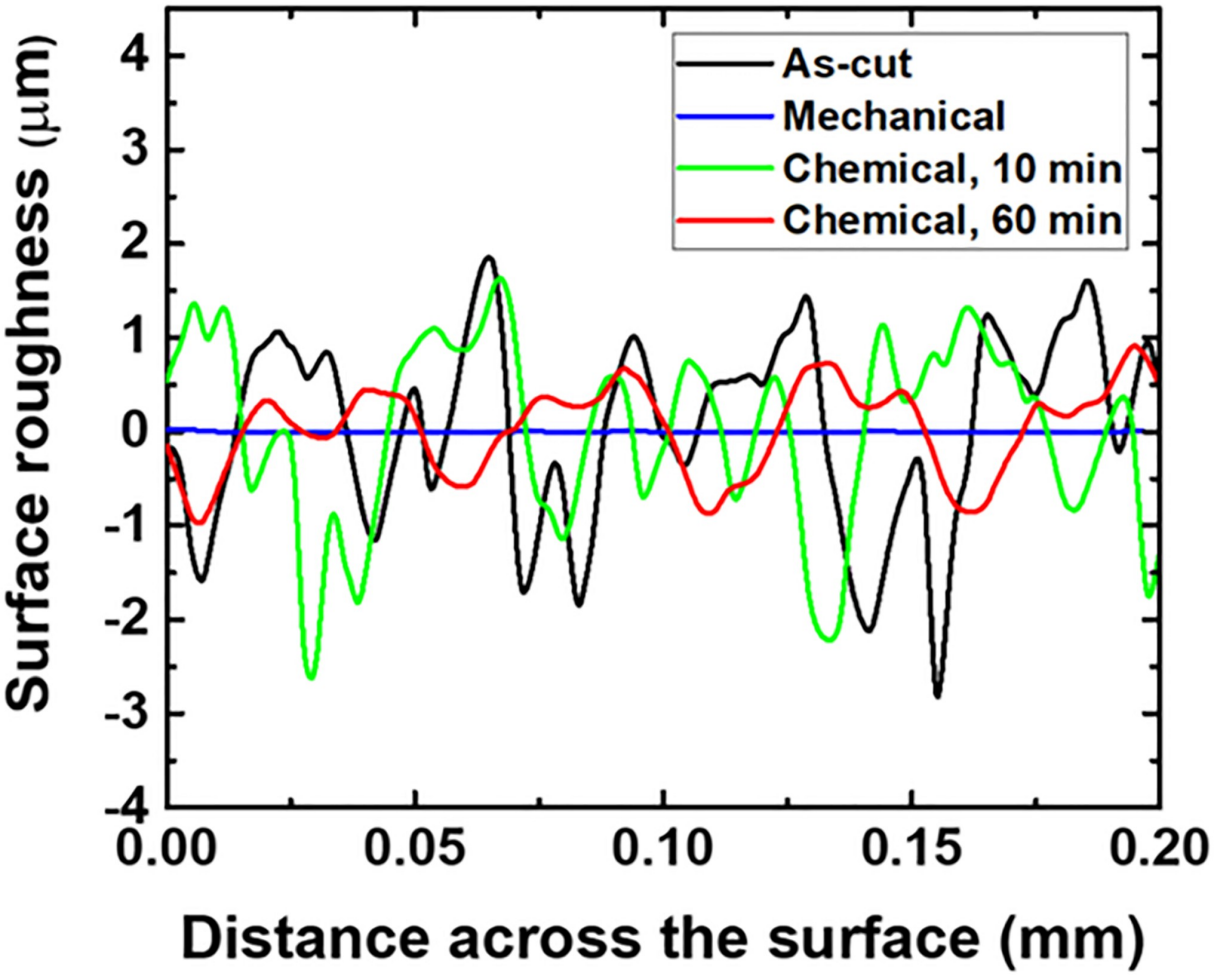
Surface roughness measurements of the 5 × 5 mm^2^ Ce:GAGG face: (a) as-cut (black), (b) mechanically polished (blue), (c) chemically polished for 10 min (green), and (d) chemically polished for 60 min (red).

Both qualitative and quantitative analyses of the surface roughness can reveal the effectiveness of the chemical polishing treatment, as shown in Fig 5 and Table 2. Evidently, the initial roughness of the as-cut sample was 928 nm, which decreased to 430 nm after chemical polishing for 60 min. In addition, the degree of chemical etching varied significantly with the polishing time, and there existed an optimal etching time that produced the desired surface for a higher light output.

**Table 2:** Comparison of the surface roughness and waviness values obtained after the different surface treatments.

Sample	R_a_ (nm)	W_a_ (nm)
As-cut	928 ± 10.6	1131 ± 12.4
Mechanical	7.5 ± 1.6	35 ± 3.5
Chemical, 10 min	764 ± 7.9	951 ± 10.7
Chemical, 60 min	430 ± 6.9	512 ± 7.8
Chemical, 90 min	422 ± 7.2	501 ± 8.0
Chemical, 120 min	420 ± 9.2	490 ± 11.0

* Each R_a_ and W_a_ value represents the average value and standard deviation of the randomly selected areas on the crystal.

** W_a_ values represent the degree of surface irregularities with a spacing greater than the surface roughness.

### Energy spectrum evaluation

The relative light output and energy resolution of the 5 × 5 × 2 mm^3^ Ce:GAGG single-crystal samples for the different surface treatments are listed in Table 3. As expected from the SEM images shown in Fig 4, the mechanically polished sample showed the highest increase of 38.3% in the relative light output and an improvement of 3.4% (absolute value) in the energy resolution. For the chemically polished samples, the largest enhancement in the light output was acquired after chemical polishing for 60 min, beyond which a slight degradation in the scintillator performance was observed.

**Table 3:** Relative light output and energy resolution of the 5 × 5 × 2 mm^3^ Ce:GAGG single crystals before and after the different surface treatments.

Treatment	Relative light output (relative value, %)^†^	Energy resolution before/after treatment (%)^‡^
Mechanical	138.3 ± 0.9	14.6 ± 0.3/11.2 ± 0.5
Chemical, 10 min	108.5 ± 2.5	14.7 ± 0.6/14.2 ± 0.6
Chemical, 20 min	110.7 ± 1.7	14.4 ± 0.5/13.3 ± 0.2
Chemical, 30 min	113.6 ± 2.9	15.1 ± 0.2/13.5 ± 0.3
Chemical, 60 min	133.1 ± 1.1	15.1 ± 0.2/12.7 ± 0.1
Chemical, 90 min	131.6 ± 1.6	14.7 ± 0.2/12.8 ± 0.2
Chemical, 120 min	130.3 ± 1.8	14.9 ± 0.3/13.2 ± 0.5

^†^Relative light output was calculated as the average of three different sample outputs and their standard deviations.

^‡^Data are the average values and standard deviations of the resolutions exhibited by three samples before (baseline) and after the treatments.

**Fig 6:**
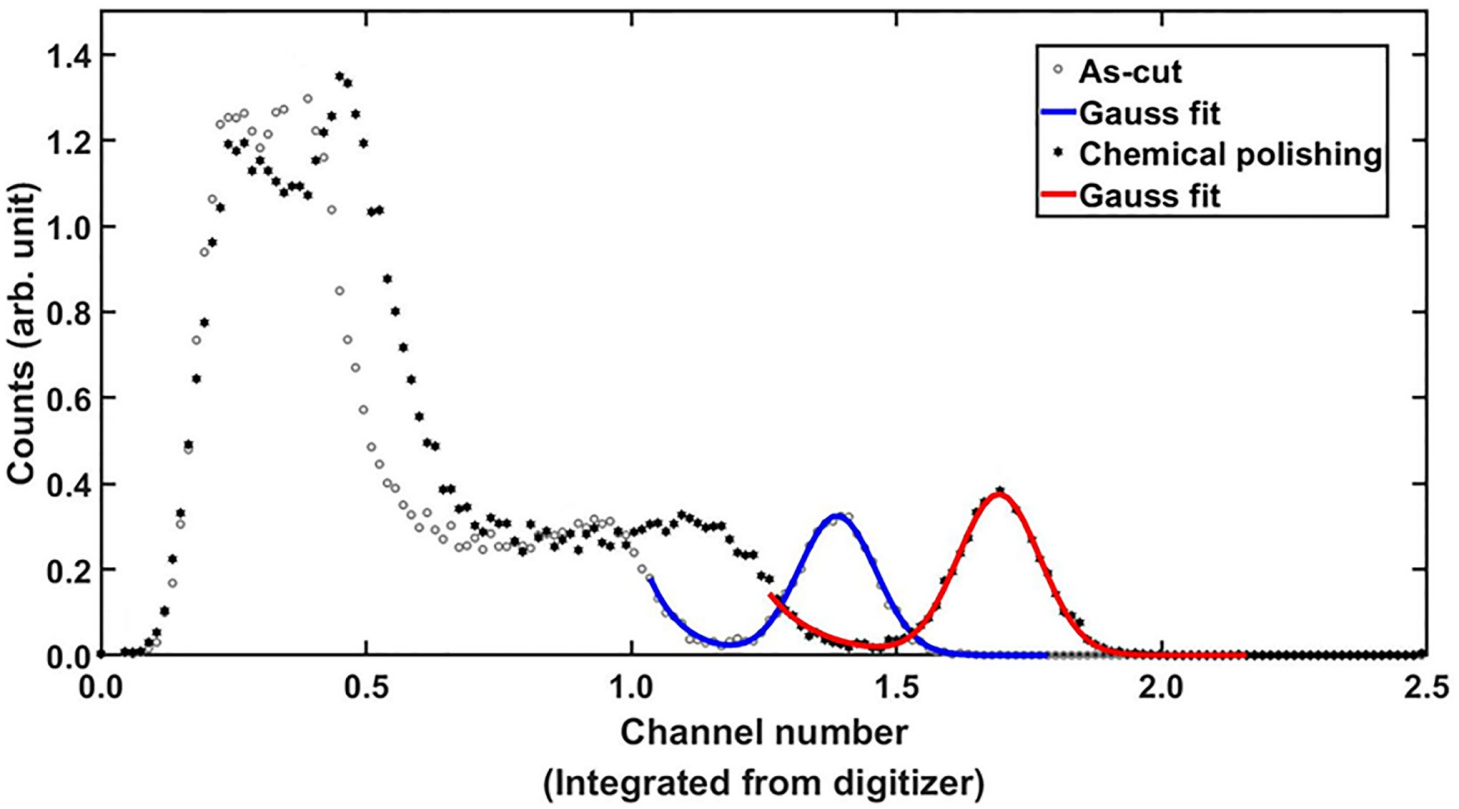
Example of pulse height spectra of a 5 × 5 × 2 mm^3^ Ce:GAGG single-crystal sample, coupled to a PMT, before and after chemical polishing: (a) as-cut and (b) chemically polished for 60 min. A ^137^Cs radioactive source was used to obtain the pulse height spectra.

The 5 × 5 × 2 mm^3^ Ce:GAGG single crystals, chemically polished for 60 min, demonstrated a 33.1% increment in the relative light output and 2.4% improvement in the full width at half maximum (absolute value), i.e., energy resolution, as shown in Fig 6. The reason for why the longer chemical treatment time did not produce any substantial improvement in the light output was not perfectly clear; however, it is likely that the long duration chemical treatment led to an excessive change in the crystal surface morphology that was counteractive to light extraction.

**Fig 7:**
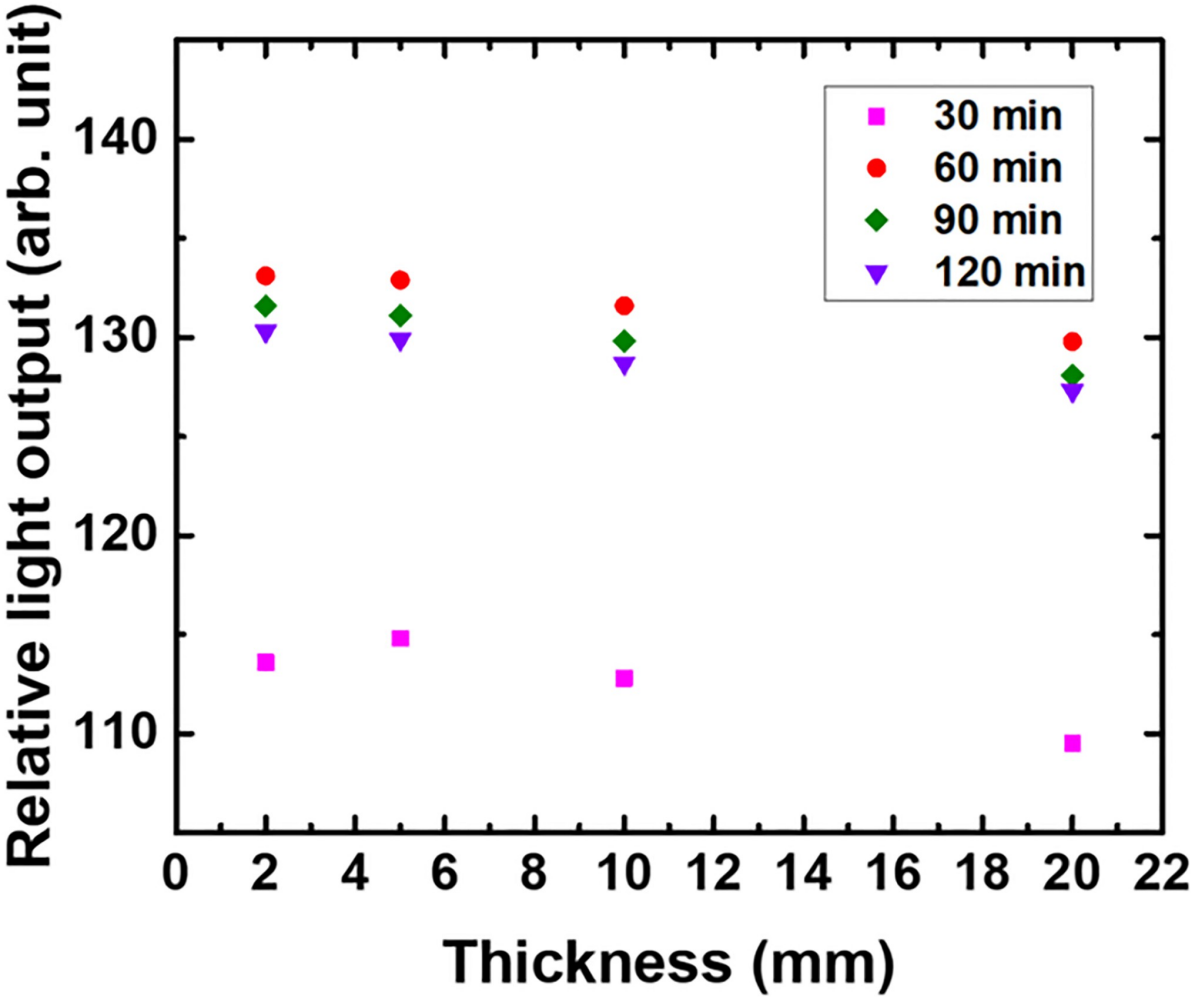
Relative light output compared to itself (as-cut) after chemical polishing for 30, 60, 90, and 120 min for various Ce:GAGG crystal thicknesses.

The scintillation light photons produced in a crystal are emitted isotropically, and thus, their propagation is affected by the surface roughness that determines the reflectivity and reflection angle. This, in turn, determines the number of reflections and the light passage travelled by a light photon before being detected by the photosensor [26]. This geometrical influence on the light output was evaluated by changing the thicknesses of the Ce:GAGG crystals to 2, 5, 10, and 20 mm, as shown in Fig 7, which shows the changes in the relative light outputs before (as-cut baseline) and after chemical polishing with various crystal thickness. In all the cases, a chemical polishing time of 60 min was optimal with respect to the light output. The slight decrease in the slope with the increasing crystal thickness corresponds to the geometrical effect [13]. In addition, after chemical polishing for 60 min, 6.3% of the crystal was etched away, whereas 11.2% of the crystal was etched away after 120 min of chemical polishing. This indicates that the optimal chemical polishing time for the Ce:GAGG crystal is 60 min under the aforementioned treatment conditions.

## Conclusions

It is known that improving surface morphology (smoothing) can help improve light transportation to a photodetector (optical transportation property) [12, 20]. Moreover, surface treatment such as chemical or mechanical polishing can improve crystallinity through reduction of surface defects such as dangling bonds (luminescent property). While Both factors (morphology and crystallinity) can contribute to improving the light outputs of the samples, it is difficult to isolate the effects of the two factors.

In this study, we demonstrated the effect of surface treatment on the luminescence and optical properties of Ce:GAGG crystals, chemically polished with phosphoric acid, from the crystallographic perspective. The obtained PL spectra indicated that the luminescence properties were strongly related to the surface morphology and crystallographic structure of the crystals. The existence of surface defects such as dangling bonds on the rough surface resulted in surface recombination and subsequent non-radiative emissions, which reduced the PL signal intensity in rough as-cut crystals. However, chemical polishing for 60 min significantly reduced the number defects such as dangling bonds which suppressed the non-radiative processes and enhanced the light output.

Mechanical polishing produces the flattest surface leading to the highest light output. Chemical polishing was verified to produce a light output improvement comparable to that produced by mechanical polishing, with an optimal dipping time of 60 min in phosphoric acid at 190°C, for the Ce:GAGG crystals, and can be attributed to improvements in morphology and crystallinity. Dipping times beyond 90 min produced negligible improvement in the performance of the scintillator and caused an excessive material loss. These results indicate that chemical polishing can be an attractive alternative to mechanical polishing, particularly when complex scintillator shapes are involved or for large-scale operations.

## Supporting information

S1 Fig SEM images (5 μm scale) of the 5 × 5 × 2 mm^3^ Ce:GAGG single crystals: (a) as-cut, (b) mechanically polished, and (c)–(f) chemically polished for 10, 30, 60, and 120 min.
(TIF)

S2 Fig SEM images (200 μm scale) of the 5 × 5 × 2 mm^3^ Ce:GAGG single crystals: (a) as-cut, (b) mechanically polished, and (c)–(f) chemically polished for 10, 30, 60, and 120 min.
(TIF)
