# Supplementary figures and images for: Scintillation characteristics of chemically processed Ce:GAGG single crystals
Source: PLoS One. 2023 Mar 7;18(3):e0281262. doi: 10.1371/journal.pone.0281262 (PMC9990913; doi:10.1371/journal.pone.0281262)

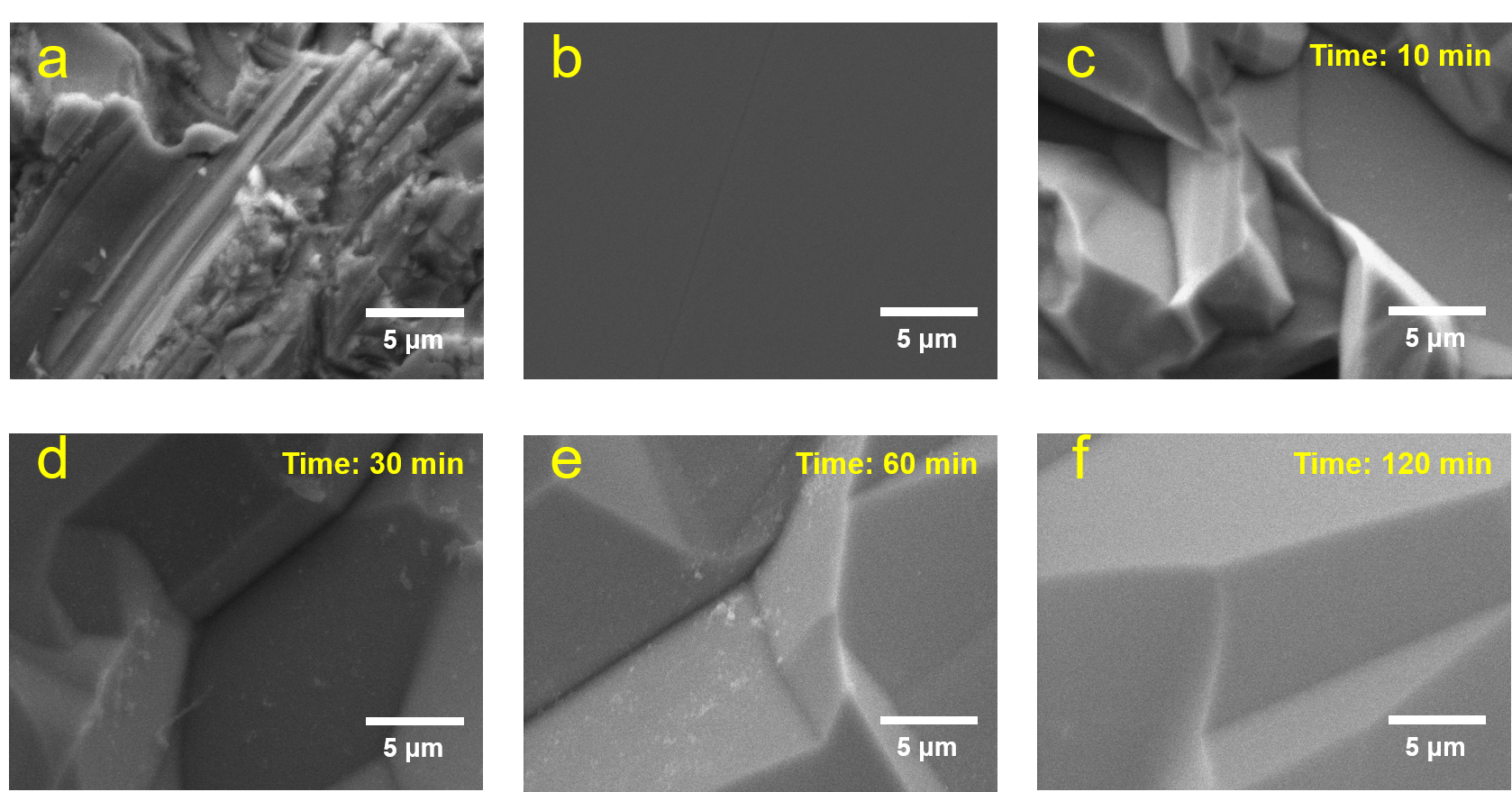

Supplement: S1 Fig — SEM images (5 μm scale) of the 5 × 5 × 2 mm3 Ce:GAGG single crystals: (a) as-cut, (b) mechanically polished, and (c)–(f) chemically polished for 10, 30, 60, and 120 min. (TIF) [file pone.0281262.s001.tif]

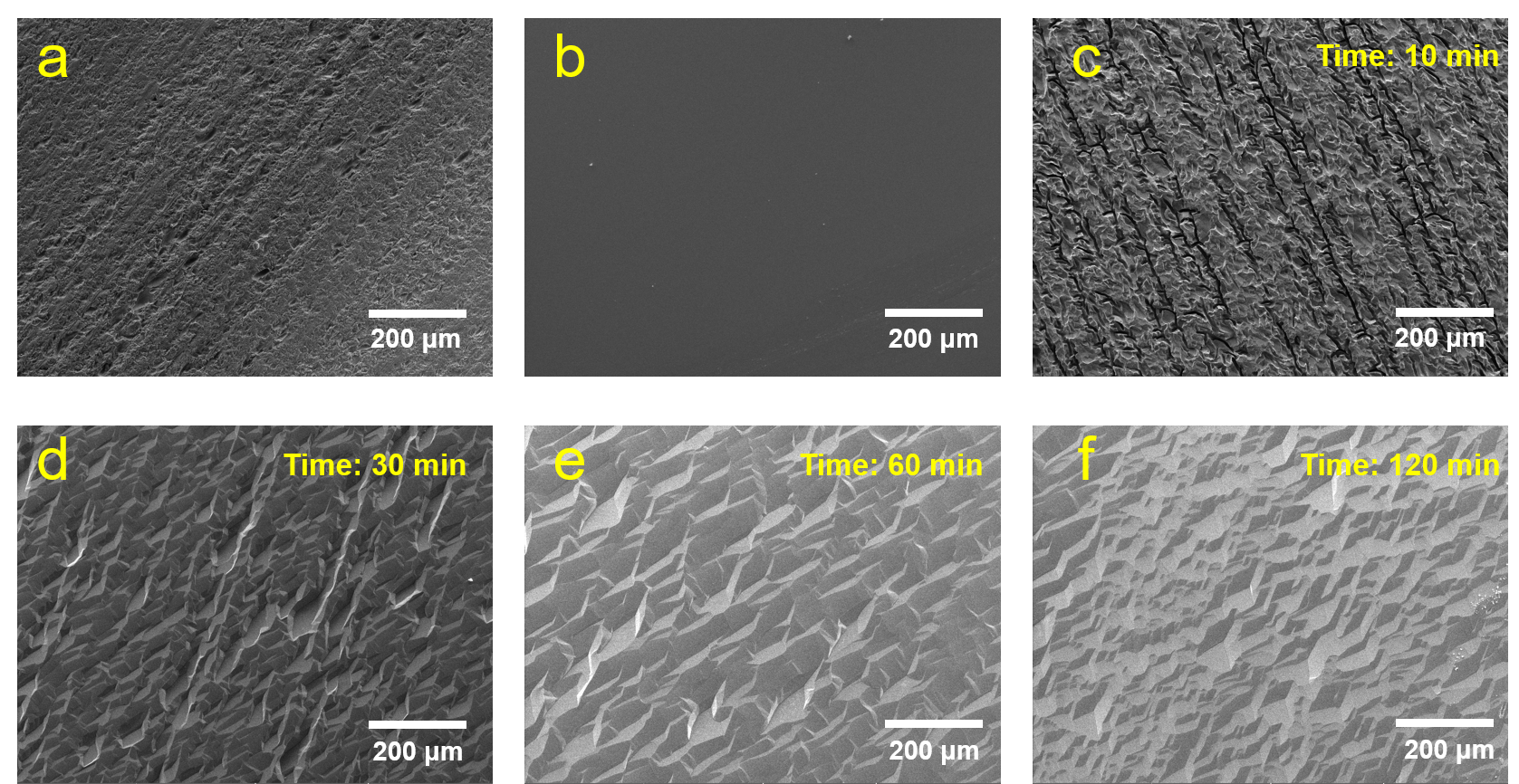

Supplement: S2 Fig — SEM images (200 μm scale) of the 5 × 5 × 2 mm3 Ce:GAGG single crystals: (a) as-cut, (b) mechanically polished, and (c)–(f) chemically polished for 10, 30, 60, and 120 min. (TIF) [file pone.0281262.s002.tif]
